# Molecular identification, characterization, and antagonistic activity profiling of *Bacillus cereus* LOCK 1002 along with the in-silico analysis of its presumptive bacteriocins

**DOI:** 10.5455/javar.2022.i635

**Published:** 2022-12-31

**Authors:** Samarth Islam, Mithila Farjana, Muhammad Ramiz Uddin, Sharmin Akter, Anika Jabin, Hazika Tuz-Zohura Nafisa, Siam Siraji, A K M Helal Morshed, Fahmida Hoque Rimti, Zannatul Naim, Mohiuddin Sakib, Pallab Sarker, Sabiha Naznin, H.M. Iftekhar Alam, Tanzila Ismail Ema, Mahbuba Siddiquy, Mohammad Habibur Rahman

**Affiliations:** 1 Department of Biochemistry and Microbiology, North South University, Dhaka, Bangladesh; 2 Department of Chemistry and Biochemistry, University of Oklahoma, Norman, OK, USA; 3 Department of Biology, Indiana State University, Terre Haute, IN, USA; 4 Department of Microbiology, University of Dhaka, Dhaka, Bangladesh; 5 Pathology and Pathophysiology Major, Academy of Medical Science, Zhengzhou University, Zhengzhou, China; 6 Bachelor of Medicine and Bachelor of Surgery, Chittagong Medical College, Chattogram, Bangladesh; 7 Department of Animal Production and Management, Sher-e-Bangla Agricultural University, Dhaka, Bangladesh; 8 Department of Medicine, Sher-E-Bangla Medical College Hospital, Dhaka, Bangladesh; 9 Department of Biomedical Engineering, Military Institute of Science and Technology, Dhaka, Bangladesh; 10 Division of Validation, BEXIMCO Pharma Ltd., Dhaka, Bangladesh; 11 State Key Laboratory of Food Science and Technology, Jiangnan University, Wuxi, China; 12 Vaccinology Lab, Department of Microbiology and Hygiene, Bangladesh Agricultural University, Mymensingh, Bangladesh; 13 Immunoinformatics and Vaccinomics Research Unit, RPG Interface Lab, Jashore, Bangladesh

**Keywords:** *B. cereus* LOCK 1002, 16S rRNA analysis, PEMBA medium, cerein 7B, bacteriocins, genetic clustering, antimicrobial activity profiling of *B. cereus*

## Abstract

**Objectives:**

This research aimed to isolate, identify, and characterize a new strain of *Bacillus cereus* through different molecular biology approaches so that it could be further studied for therapeutic purposes against selective enteric pathogens.

**Materials and Methods:**

Pure isolates of *B. cereus* were prepared from buffalo yogurt samples in REMBA medium. Initially, the morphological, physiological, and biochemical properties were studied accordingly. Following the tests, the molecular identification for the strain identification was conducted through plasmid DNA extraction, PCR, agarose gel electrophoresis, and 16S rRNA sequencing up to 1.37 kb. Afterward, the antibiotic sensitivity [Epsilometer test (E-Test)] and antifungal activity were tested considering different concentrations. Being classified from the aforementioned tests, a comprehensive antimicrobial activity test was conducted using the cell-free-supernatant (CFS) of the test strain against selective enteric pathogens in humans *in vitro*. Besides, the different clusters of genes were identified and characterized for understanding the presumptive bacteriocins present in the CFS of the strain *in silico,* where molecular string properties were calculated. Finally, the evolutionary relationship among diversified bacteriocins synthesized by different *Bacillus* strains was studied to predict the CFS-containing bacteriocins of the new strain.

**Results:**

Purified isolates of *B. cereus* were Gram-positive rods and showed significant tolerance (*p *< 0.0001) to different concentrations of pH, phenol, bile salt, and NaCl. 16S rRNA revealed the strain as LOCK 1002, which was strongly sensitive to all the antibiotics used and resistant to selective antifungal agents. The CFS of *B. cereus* LOCK 1002 was found to be a very promising antagonist to all the enteric pathogens used in the culture condition. Two gene clusters were predicted to be interconnected and responsible for different presumptive bacteriocins.

**Conclusion:**

The newly identified LOCK 1002 can be a very potent strain of *B. cereus* in use as an antimicrobial agent for having different bacteriocin coding gene clusters.

## Introduction

*Bacillus cereus* is a spore-forming, gram-positive bacteria capable of infecting multiple hosts and being ubiquitous [1]. *Bacillus cereus* was found to have a remarkable tolerance to heavy metals, bile salts, and pH up to 8 [2]. It typically has a high occurrence in dairy products and is well known for the spoilage of pasteurized milk, leading to foodborne illness [3]. As concerning as it is, multiple foodborne diseases and typically severe clinical conditions have been affiliated with *B. cereus* as a causative agent [4]. Especially prominent in foodborne illness, two distinct infectious characteristics (emetic or diarrheal syndrome) are linked with the release of particular potent peptide toxins and proteinaceous enterotoxins [5]. It may seem as though *B. cereus* is renowned for its potential as an enteropathogenic agent [6]. However, concurrent studies have elucidated that evolving strains of *B. cereus*, with the help of differing expansion and interchange of genetic material in the specific *Bacillus *gene, result in the formation of newly emerged anthrax-like clinical manifestations in mammals, especially in humans [5]. As prevalent as gastrointestinal infections can be, clinical studies demonstrated that *B. cereus* induces bloodstream infections, with patients exhibiting nosocomial infections [6]. Besides local and systemic infections, *B. cereus* in an immunocompromised individual generates multi-characteristic clinical conditions such as endophthalmitis, endocarditis, meningitis, septicemia, and encephalitis, leading to 10% of patient deaths [7]. Surprisingly, due to their ability to withstand the surrounding environmental stresses of high temperature, acidic-gastric environment, and moisture, several *Bacillus *species are now being utilized as probiotics. Some commercially applied *Bacillus *species are used industrially: *B. cereus*, *B. subtilis*, B. clausii, B. coagulans, *B. polyfermenticus*, *B. licheniformis*, and *B. pumilus*. Several *Bacillus *species show significant antimicrobial, antioxidant, anticancer, and vitamin-producing characteristics when supplemented [8]. Compared to the B. clausii and *B. subtilis*, selective *B. cereus* strains can survive longer by attaching their hydrophobic spores to the epithelial cells, which shows prominent therapeutic attributes against gut pathogenic inhabitants including- *Salmonella*, *H. pylori*, and *E. coli* [9]. Potential therapeutics that include prevalence analysis, virulence genes, antimicrobial susceptibility tests, and gene cluster analysis for bacteriocins are being tested to combat the rampant emergence of *B. cereus* infections [10]. An ever-increasing antibiotic resistance, shaped most evidently by environmental factors, is currently being assessed with the utmost strategies [11].

Bacteria are incubated in Lysogeny broth at 37°C (24 h), and 10-min centrifugation (3,000 rpm) is carried out to detach cell-free-supernatant (CFS) from cell debris. In order to get a substantially CFS, this process is performed twice [12]. According to a recent study, the CFS from *Lactobacillus plantarum* NIBR97, which contains nevel bacteriocins, may potentially serve as a natural disinfectant substitute for chemical ones [13]. Another study tested the potential of the CFS extracted from liquid cultures of particular local lactic acid bacteria (LAB) and yeast isolates [14]. Recent studies revealed that microorganisms like *Salmonella* spp., *L. monocytogenes*, and *S. aureus* can be inhibited by CFS *in vitro* in both the co-culture and delayed culture methods [14]. CFS from the LAB isolates is reported as a strong inhibitor of enteric pathogens when co-cultured. The inhibitory activity of CFS depends primarily on the existence of active substances secreted into the supernatant. Active microbial metabolites, including bacteriocins, which are generated and secreted by bacteria into the growth medium, can inhibit Gram-positive and Gram-negative pathogens [14]. Bacteriocins are ribosomally manufactured, post-translationally modified molecules that do not affect the producer strain but can kill closely related bacteria [15]. The effectiveness of the CFS mainly depends on the source strains, toxicity level, and proteinaceous profiles [16]. The cationic bacteriocin exploits the anionic microbial cell wall by forming ion-selective pores; thus, the permeability increases and the microbes fail to survive [17]. A recent study identified 16S rRNA from numerous *B. cereus* strains during their gene sequencing. Their bacteriocinogenic production activity was shown in culture conditions against four different microbes, including *S. aureus, E. coli, B. cereus, *and *Salmonella* spp. [18]. A few bacteriocins have been approved for preclinical applications, such as mutacin 1,140, microbisporicin, and duramycin; due to their significant activities against *Clostridium difficile* infection, multiresistant pathogens, and cystic fibrosis, respectively [19]. Hence, pursuing novel and promising bacteriocins for clinical and therapeutic applications has become a significant demand in the future.

Considering the aforementioned information, this current research aimed to isolate a new *B. cereus* strain through 16S rRNA analysis, following which morphological, physiological, and biochemical characterizations were conducted. Afterward, the antibiotic and antifungal sensitivity were tested before subjecting the test strain to its antimicrobial profiling in response to selective enteric pathogens. Finally, systems biology-based predictions of the presumptive bacteriocins and the genes responsible for those antimicrobial protein (AMPs) networks were studied.

## Materials and Methods

### Isolation of B. cereus

In this study, a total of 43 milk samples were collected from buffalo yogurt and inoculated on polymyxin pyruvate egg-yolk mannitol–bromothymol blue agar (PEMBA) medium, which is selective for growing *B. cereus* [2,18]. After successive subcultures, pure isolates were stored at −20°C with 50% PBS-glycerol [20].

### Morpho-physiological and biochemical characterization

The isolated *B. cereus* was characterized morphologically by its colony size, shape, and Gram staining, and physiologically by analyzing its growth at high pH, phenol, bile salts, and NaCl [18]. Besides, the biochemical characterization was conducted by catalase, coagulase, Voges-Proskauer, indole, urease, nitrate reductase, β-Galactosidase, and H_2_S production tests [21].

### Molecular analysis of the B. cereus strain

The strain was identified by sequencing the *16S rRNA* gene. The ribosomal RNA was extracted and amplified using the Veriti® 99 well Thermal Cycler PCR system (Model No. 9902) with the “Universal 16S rRNA Specific Primer” [18]. Following the observation of the desired PCR amplicon, it was purified enzymatically for subjecting to the “Sanger sequencing.” The bi-directional sequencing was conducted by using the BDT v3.1 Cycle Sequencing Kit on the ABI 3,730xl Genetic Analyzer, where 8F and 1,401R primers were used [2,18]. Afterward, the consensus sequence was uploaded in NCBI’s FASTA format through Gene Bank, and an accession number for the targeted strain was received. Finally, the phylogenetic tree was constructed to determine the evolutionary relationship of the strain of *B. cereus*. The qualitative relationship study was conducted by MEGAX [22], whereas the quantitative relationship measurement was determined using Phylogeny.fr [23].

### Antibiotic and antimycotic sensitivity profiling

The antibiotic sensitivity test of the identified *B. cereus* strain was carried out by epsilometer, where the MIC_50_ and MIC_90_ were tested, considering distinguishable minimum inhibitory concentrations (MIC) (μg/ml) of different antibiotics [24]. On the other hand, selective antifungal agents were used in different concentrations as the antimycotic agents, namely, viodine (%, *v*/*v*), polydine (%, *w*/*v*), nystatin (%, *v*/*v*), and fluconazole (%, *w*/*v*) [25] to assess the sensitivity of the *B. cereus* strain.

### Antimicrobial activity profiling of the B. cereus strain

Firstly, the CFS of the overnight pure culture broth was collected through centrifugation at 15,000 rpm (22,388 g) using a refrigerated centrifuge machine (NEUATION; Model No. UC02R) for ensuring maximum output [26]. The pure supernatant was placed into a nutrient agar medium to have a stick-plate co-culture with two different enteric pathogens in each petri dish at a time [27]. In that case, the CFS was kept in the middle portion, while the two enteric pathogens were seeded at the two opposite ends of the Petri plate. Every 24 h for 3 days, the pathogenic proliferation patterns near the CFS margins were measured (mm) [18].

### In silico analysis of the presumptive bacteriocins of the B. cereus CFS

Several bacteriocins are biosynthesized naturally by *B. cereus* depending on their culture condition and metabolic features. In this study, several *B. cereus*-producing bacteriocins have been obtained from the STRING database of protein networking. Using Cytoscape 3.8.2 (https://cytoscape.org/), the bacteriocin network’s nodes and edges were detected and characterized [28], which is operated via the Java Runtime Environment. Besides, the genes that code for those bacteriocins in *B. cereus* strains have been identified and characterized using the BACTIBASE [29] and GeneMANIA databases [30]. Finally, the bacteriocin genes of *B. cereus* strains were analyzed based on their nodes of string formation, regions on the chromosome; gene-fusion (GF); phylogenetic co-occurrence (PO), coexpression (CO), homology (H), experimentally defined interactions (EI); database annotation (DA), automated text mining (AT), and the total scores. The clustering of the interactive bacteriocin nodes of *B. cereus* was constructed with the tools Morpheus [31] and Heatmapper [32]. A complete linkage clustering of proteins with the Euclidean distance measurement method was used to assess the *B. cereus* bacteriocins. The interaction source of the bacteriocin nodes of *B. cereus* strains was identified using Cytoscape 3.8.2 [29]. Phylogenetic analysis of several *B. cereus* bacteriocin genes were accomplished to determine their quantitative divergence from each other via Phylogeny.fr [23] and Clustal Omega [33]. The protein clustering was conducted following the parameters: nodes of string formation; neighborhood on the chromosome; GF; PO; H; CO; EI; DA; AT; and the combined scores (CO) [32].

### Statistical analysis

The statistical survivability assessment (Tukey’s multiple comparison test) of the tested *B. cereus* strain based on the spectrophotometric (UV-1280, Shimadzu) optical density values resulted in 650 nm (OD_650_; 72 h) was conducted by “Rstudio” (R-4.0.2) [34–38], and GraphPad Prism (V. 8.0.1) [39–44].

## Results

### Morpho-physiological and biochemical characterization

In this study, 11 *B. cereus* isolates were purified, and the isolates exhibited medium-sized (5–7 mm) whitish colonies on PEMBA, and in Gram stain, the cells were found Gram-positive rods. The isolates were tolerant to a broad range of pH (5.0–9.0), phenol (0.3%), bile (0.3%), and NaCl (3%). The presumptive isolates in the biochemical analysis revealed positive reactions for catalase, coagulase, nitrate reductase, and Voges-Proskauer tests, but variable (+/−) results for the urease test. Table 1 shows that it was negative for galactosidase, H_2_S production, and indole reaction tests.

**Table 1.** Morphological, physiological, and biochemical characteristics of *B. cereus* LOCK 1002.

	Properties
Morphological	
Size	5–7 mm
Color	Whitish colonies
Motility	Motile
Physiological	
pH tolerance	pH(5.0–9.0)
Phenol tolerance	0.3%
Bile tolerance	0.3%
NaCl tolerance	3.0%
Biochemical	
Gram staining	+
Catalase	+
Coagulase	+
Voges-Proskauer	+
Urease	Variable (+/−)
Nitrate reductase	+
H_2_S production	-
Indole	-
β-Galactosidase	-

*p *< 0.0001 (α = 0.05) resulted from the physiological tests at OD_650_.

### Molecular identification for strain selection

**Figure 1.**
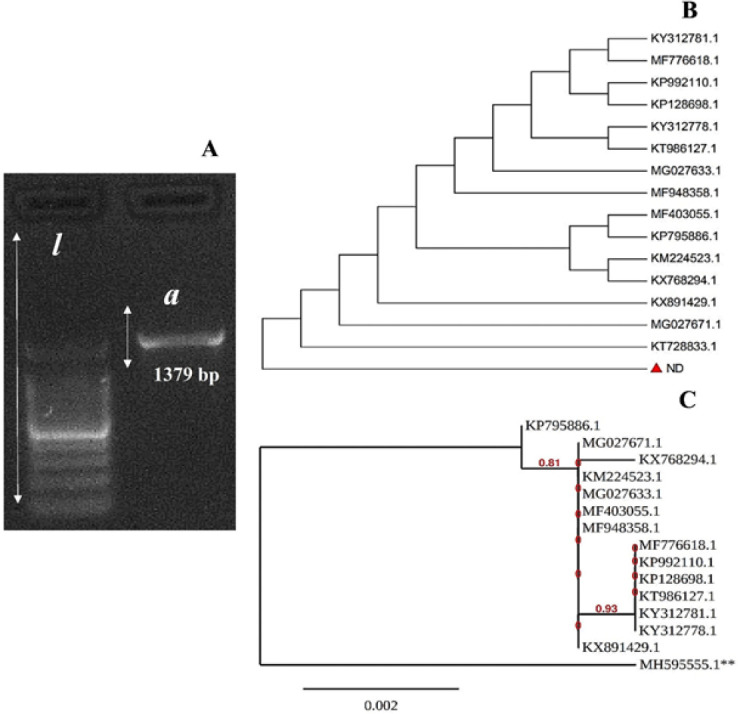
The illustration of the agarose gel electrophoresis of the 16S rRNA amplicon (A); the evolutionary relationship considering both qualitative (B) and quantitative (C) means of *B. cereus* LOCK 1002.

In PCR amplification of the *16S rRNA* gene, a 1,379-bp amplicon of *B. cereus* was found compared to a 2-kb ladder (Fig. 1A). Following PCR purification and Sanger sequencing of the* 16S rRNA* gene, a consensus sequence was retrieved and uploaded into NCBI through Gene Bank, which confirmed that the strain was LOCK 1002 (accession number MH595555.1). 

The phylogenetic tree encompasses a total of fifteen distinct bacterial strains of *B. cereus* LOCK 1002 (Fig. 1B). The target strains’ higher levels of resemblance to the reference strains discovered in the GenBank allowed for the completion of their identification. There was an evolutionary lineage of *B. cereus* LOCK 1002 was found with the other *Bacillus strains*, including- KP795886 (*Bacillus* sp. Bac389R), MG027671.1 (*B. cereus* strain VBE16), KX768294.1 (*Bacillus* sp. strain P12), KM224523.1 (*Bacillus* sp. XQW2), MG027633.1 (*B. cereus* strain VBE12), MF403055.1 (*Bacillus* sp. strain ZSQ1), MF948358.1 (*Bacillus sp*. strain FJAT-29907), MF776618.1 (*Bacillus* sp. strain MBL_B21), KP992110.1 (*Bacillus* sp. BG3-7), KP128698.1 (*Bacillus thuringiensis* strain Ou2), KT986127.1 (*Bacillus thuringiensis* strain Lmb062), KY312781.1 (*B. cereus* strain GT48), KY312778.1 (*B. cereus* strain GT36), and KX891429.1 (*Bacillus* sp. strain PG2). At the same time, KP795886 (*Bacillus* sp. Bac389R), MG027671.1 (*B. cereus* strain VBE16), and KX768294.1 (*Bacillus* sp. strain P12) were identified as the three significantly distinct strains of *B. cereus* that shared the most similarities with *B. cereus* LOCK 1002 (Fig. 1C). Both *B. anthracis* LOS6 and *B. thuringiensis* Ou2 were closely linked to those bacteria that belonged to distinct species. On the other hand, *B. cereus* LOCK 1002 and KT986127.1 (*Bacillus thuringiensis* strain Lmb062) have a remote link to each other (Fig. 1B and C).

**Table 2.** Antibiotic sensitivity profiling of *B. cereus* LOCK 1002.

Antibiotics	Method^a^	*Bacillus cereus LOCK 1002*		
		Range	MIC_50_ (µg/ml)	MIC_90_ (µg/ml)
**Streptomycin**	E-test	16–58	35.5	54
**Penicillin G**	E-test	0.015–>30	31.9	32
**Cefotaxime**	E-test	0.1–>28	>21.1	≥28
**Erythromycin**	E-test	0.035–3.04	0.091	1.81
**Amphotericin**	E-test	0.03–1	0.523	0.775
**B**				
**Tetracycline**	E-test	0.083–29	1	6.26
**Vancomycin**	E-test	1–16	4	8.1
**Gentamicin**	E-test	0.094–≥0.80	0.5	≥0.79
**Amoxicillin**	E-test	1.6–15	8.1	16.09

a Epsilometer test.

^b^Minimum concentration for 50% inhibition (MIC_50_).

^c^Minimum concentration for 90% inhibition (MIC_90_).

### Antibiotic and antimycotic sensitivity test

**Figure 2.**
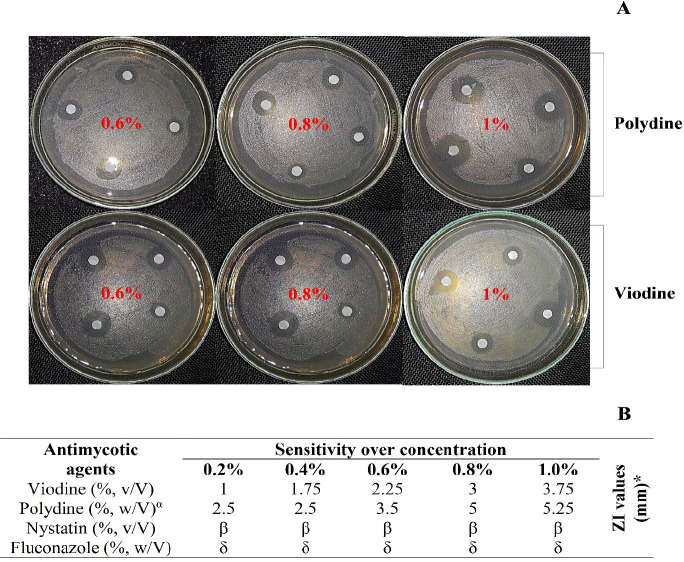
Antifungal profiling of *B. cereus* LOCK 1002 considering different concentrations. The qualitative and quantitative assessments are represented in A and B respectively.

Quantitative assessment of the antibiotic susceptibility using the E-test showed significant susceptibility profiles of *B. cereus* LOCK 1002 in terms of the MIC_50_ and MIC_90_ values (Table 2). Considering the concentration and inhibition patterns, erythromycin was found to be the best-performing drug, with the lowest MIC_50_ and MIC_90_ scores of 0.091 and 1.81, respectively. In addition to erythromycin, gentamicin, amphotericin B, and tetracycline were included. In contrast, streptomycin was the least effective drug since it had the highest MIC_50_ (35.5 µg/ml) and MIC_90_ (54 µg/ml) values (Table 2). Similarly, increased antifungal sensitivity of *B. cereus* LOCK 1002 was observed with the increased concentration of viodine and polydine (Fig. 2A). Surprisingly, this strain was found strongly resistant to Nystatin and, partially resistant to Fluconazole, producing no zone of inhibition at five different concentrations (Fig. 2B), which is alarming.

### Antimicrobial activity test

**Figure 3.**
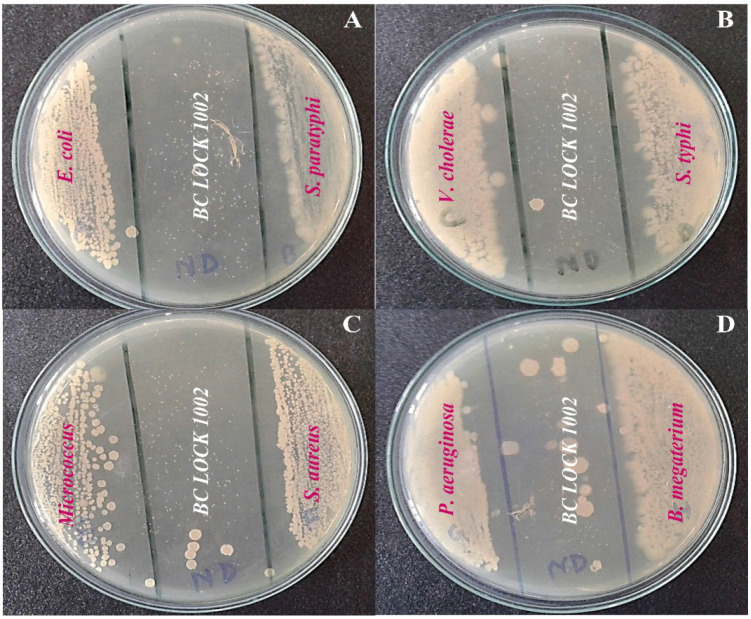
The antibacterial activity of the CFS of B.* cereus* LOCK 1002 against selective enteric pathogens of human.

The CFS of *B. cereus* LOCK 1002 showed maximum efficacy against *E. coli* with an inhibitory zone of 8 mm (Fig. 3A), whereas minimum inhibition efficiency (2 mm) was observed against *S. aureus* (Fig. 3C). The test strain’s CFS conferred significant antagonistic effects against *S. paratyphi*, *S. typhi*, *P. aeruginosa*, *B. megaterium*, *V. cholerae*, and *Micrococcus* (Fig. 3A–D). The quantitative measurement of their zones of inhibition (ZI) is also described (Table 3).

**Figure 4.**
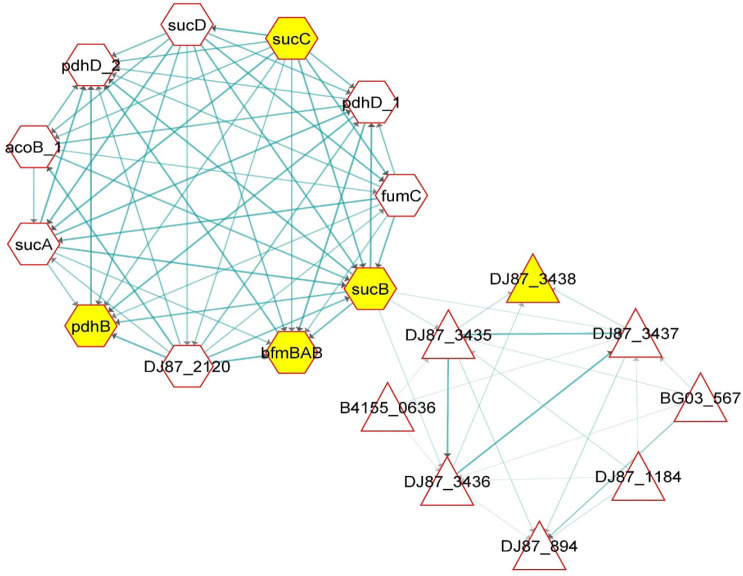
Genetic interactions behind the clustering of different presumptive antimicrobial peptides in the CFS of *B. cereus* based on their genetic STRING profiles.

**Table 3.** Antimicrobial activity of *B. cereus* LOCK 1002 to selective infectious enteric pathogens.

Name of the pathogens	Antimicrobial profiles	ZI (mm)
***Escherichia coli***	++	8
***Vibrio cholerae***	++	7
***Salmonella typhi***	++	6
***Salmonella paratyphi***	++	4
***Micrococcus***	++	6.5
***Pseudomonas aeruginosa***	++	6
***Bacillus megaterium***	++	6
***Staphylococcus aureus***	+	2

### Predicting the genetic interactions behind the clustering of functional bacteriocins to each other in B. cereus

In the string networking of the genes of *B. cereus* strains obtained from the STRING database (https://string-db.org/) and Cytoscape 3.8.2 (https://cytoscape.org/) [31], a diversified group of genes is involved in the control of biosynthesizing different AMPs (Fig. 4). The genes for encoding radical SAM protein (gene identifier: DJ87_238), cyclodehydratase domain (gene identifier: DJ87_3436), and SagB-type dehydrogenase domain protein (gene identifier: DJ873437) of *B. cereus* are more strongly interconnected to each other. On the other hand, genes responsible for synthesizing uncharacterized peptides in *B. cereus* strains are DF87_1184; DJ87_2735; and DJ87_3435 (Fig. 4). More interestingly, DJ87_3435, DJ87_3436, and DJ87_3437 possess strong co-expression profiles and act as interaction sources with each other (Fig. 4) during protein synthesis. The functional bacteriocin-coding genes were connected with some other gene clusters whose function is still undefined, such as sucB, bfmBAB, pdhB, sucC, and DJ87-3438 (Fig. 4. Among all the genes, DJ87_3435 was the strongest source-signaling gene in the protein clustering of *B. cereus* (Fig. 4), where three more uncharacterized proteins were observed (*, ***, ##) forming significant clusters with a radical SAM protein (**). In contrast, the cyclodehydratase domain (###) and SagB-type dehydrogenase domain protein (β*) are strongly interconnected to each other but distantly related to the other protein clusters (Fig. 4). These findings indicate that many unknown bacteriocins of *B. cereus*, including the LOCK 1002 strain, have yet to be profiled.

**Figure 5.**
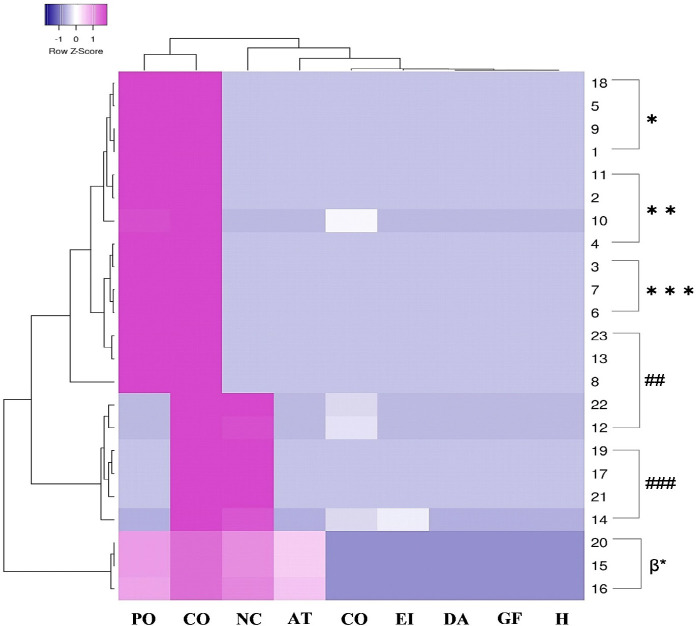
Protein clustering analysis of *B. cereus *displaying DJ87_3435 as the strongest source-signaling gene where some other uncharacterized proteins were identified as well.

**Figure 6.**
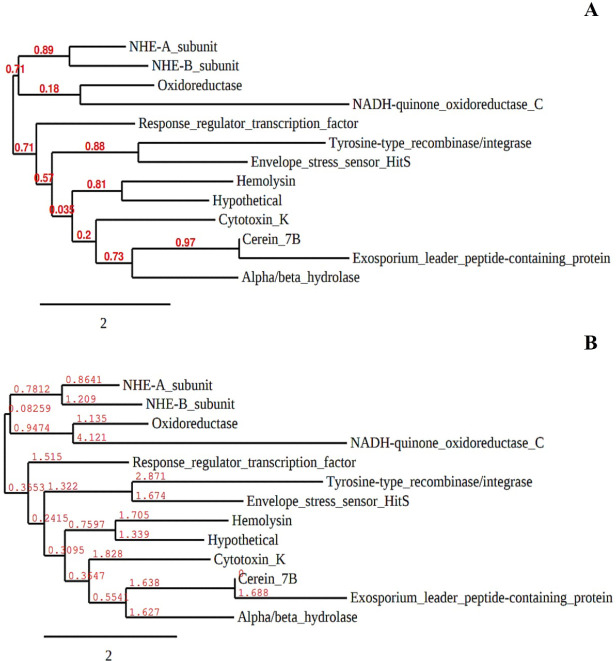
The ancestral relationship of different *B. cereus* bacteriocins with the cerein 7B depending on their major (A) and minor (B) phylogeny scores to each other.

### Evolutionary relationship of the established B. cereus toxins with the other peptides

It has been observed that the most common bacteriocin of *Bacillus* strains “cerein 7” possesses a phenomenal relationship considering its genetic makeup with some other *Bacillus* bacteriocins (Fig. 6); among which α/β hydrolase; cytotoxin K; hemolysin; oxidoreductase; tyrosine-type integrase; and NADH-quinone-oxidoreductase-C are evolutionary connected (Fig. 6A). In contrast, NHE-A, NHE-H, and response-regulator-transcription factor proteins are distantly related to the cerein 7 bacteriocins of *B. cereus* (Fig. 6B).

## Discussion

Bacteriocins are a group of ribosomally synthesized proteins of different distinguishable microorganisms that have manifested significant antimicrobial activities against selective enteric pathogens [45]. More interestingly, many of these bacteriocin-producing microbes are considered foodborne pathogens, especially the strains of *B. cereus* [46]. In recent times, many of these pathogenic microbes have been considered clinically significant due to their therapeutic-potential peptides, which can be substituted for conventional antibiotics for clinical resistance-free applications [47]. Considering, the AMPs from different strains of *Bacillus* sp. are used as potential next-generation antibiotics substitutes as revealed in different recent research programs [48], where many of them show very functional properties even under stressful circumstances such as rude temperatures, the existence of chemical substances and pH [49]. Considering the aforementioned information, this study aimed to deal with the novel bacteriocin-producing *B. cereus*, isolated from buffalo milk through comprehensive subcultures and characterization.

### Morphological & physiological observation

In the present research, morphological observations based on gram staining indicated that LOCK 1002 isolates are Gram-positive, rod-shaped bacteria that form a medium-sized, milky white colony. The isolate LOCK 1002 is motile, catalase, coagulase, and nitrates reductase positive; while showing negative reactions to H_2_S production, indole test, and β-galactosidase enzyme. The LOCK 1002 can survive a broad range of pH, Bile, Phenol, and NaCl that interpret a good tolerance level (Table 1). Similar findings were observed regarding the morphological, physiological, and biochemical features [50].

### Molecular characterization of B. cereus LOCK 1002

In a phylogenetic analysis of the *B. cereus* LOCK 1002 *16S rRNA* gene compared with a total of 15 distinct bacterial strains, there was an enhanced degree of similarity between the target strains and the reference strains in the Gene Bank. The phylogenetic profiles of *B. cereus* LOCK 1002 were compared to the sequences of KP795886 (*Bacillus spp*. Bac389R), MG027671.1 (*B. cereus* strain VBE16), KX768294.1 (*Bacillus spp*. Strain P12), KM224523.1 (*Bacillus* spp. XQW2), MG027633.1 (*B. cereus* strain VBE12), MF403055.1 (Bacillus sp. strain ZSQ1), MF948358.1 (*Bacillus sp*. strain FJAT-29907), MF776618.1 (*Bacillus sp*. strain MBL_B21), KP992110.1 *(Bacillus* sp. BG3-7), KP128698.1 (*B. thuringiensis* strain Ou2), KT986127.1 (*B. thuringiensis* strain Lmb062), KY312781.1 (*B. cereus* strain GT48), KY312778.1 (*B. cereus* strain GT36), and KX891429.1 (*Bacillus sp*. strain PG2). Figure 1C displays the three separate *B. cereus* strains that shared the most similarities with *B. cereus* LOCK 1002 and were discovered as KP795886 (*Bacillus sp*. Bac389R), MG027671.1 (*B. cereus* strain VBE16), and KX768294.1 (*Bacillus sp*. strain P12). Both *B. anthracis* LOS6 and *B. thuringiensis* Ou2 were shown to be tightly linked to bacteria from other genera. In opposition, MH595555.1 (*B. cereus* strain LOCK 1002) and KT986127.1 (*Bacillus thuringiensis* strain Lmb062) manifest a distant relationship link (Fig. 1B).

### Antibiotic and antimycotic susceptibility of B. cereus LOCK 1002

Determining antibiotic sensitivity is necessary to formulate an empirical treatment profile and effective dosage of antibiotics against a targeted organism [51]. The E-test, a novel approach developed in the late 1980s, is preferred over other conventional antibiotic sensitivity tests for its rapidity, reliability, simplicity, and versatility in its application to microorganisms and drugs. Moreover, the interpretation is simpler due to the MIC results being quantifiable [18]. Additionally, the E-test system has shown flexibility by permitting some adjustment in inoculum size, incubation temperature, and time of reading while significantly conserving the results for the isolates being tested.

In this investigation, the antibiotic susceptibility of a novel *B. cereus* strain, *B. cereus* LOCK 1002, was evaluated (Table 2), which produced results consistent with other established research [2,18]. Among the nine antibiotics used, erythromycin displayed the highest order of inhibition and inferred the greatest susceptibility of the organism. Tetracycline, gentamicin, and amphotericin B induce susceptibility against *B. cereus* LOCK 1002, albeit to a lesser degree than erythromycin, and are still considered to be in our pipeline of effective drugs. Our study displayed that the LOCK 1002 strain is resistant to streptomycin compared to the other antibiotics. This is in line with a lot of studies done in the past on the occurrence of resistance genes in *Bacillus* spp. [52]. Moreover, this study’s results directly reflect the response of *Bacillus* spp. to various antibiotics, which is similar to the results of other studies [53].

Contemporary research has also shown the antimycotic activity of *Bacillus* spp., concurrent with our research [54]. Our results revealed the existence of *in vitro* antimycotic sensitivity of *B. cereus* to antimycotic agents like polydine and viodine (Povidone-iodine), which is globally renowned for infectious disease management [55]. Even though Nystatin and Fluconazole are well-known anti-fungal agents, they were surprisingly unsuccessful in producing a zone of inhibition for our strain, even at high concentrations. When antifungal medications are administered incorrectly, such as when dosages are too low or treatment sessions are too short, resistance can emerge [56,57]. On the contrary, in the presence of polydine and viodine, ZI were observed in a dose-dependent manner at different concentrations Comparative analysis between polydine and viodine showed a substantial antimycotic action of polydine with an increasing concentration *in vitro* (Fig. 2).

### Antimicrobial activity of the CFS of B. cereus LOCK 1002

In this current research, the presence of some presumptive bacteriocins [58] was predicted in the CFS of *B. cereus* LOCK1002 based on their antimicrobial activity profiles against selective enteric pathogens [59], similar to the way probiotic microorganisms are used [60]. From peer-reviewed studies, it is deduced that several *B. cereus* strains produced novel bacteriocins, such as Cerein 7A and 8A, which exhibited antibacterial and antifungal effects against pathogenic microorganisms [61]. In this research, the CFS containing the antimicrobial peptides was found to be a very effective antagonist for *E. coli, S. aureus, S. paratyphi, S. typhi, P. aeruginosa, B. megaterium, V. cholerae*, and *Micrococcus* in the stick plate co-culture method (Fig. 3). The bacteriocins manufactured by *B. cereus* exhibit a wide spectrum of antagonistic action over Gram-positive and Gram-negative infections [62]. Following this study, antagonistic effects through the production of inhibitory zones were found against common enteric pathogens, some of which are multidrug-resistant and vancomycin-resistant. These inhibitory effects of CFS mention the existence of some presumptive bacteriocins of *B. cereus*, LOCK 1002 (Table 3). The significance of *B. cereus* has been increasing rapidly in research since the first outbreak of the COVID-19 pandemic because much-established literature strongly suggests that *Bacillus* bacteriocins can play significant roles in immunizing virus-infected individuals especially HSV, and SARS-CoV-2 [63]. Besides, it was also reported that functional AMPs can simulate the secondary immune response via successive opsonization mechanisms in the human body [64].

### *In silico* prediction of the presumptive bacteriocins present in the CFS of *B. cereus* LOCK 1002

The *in silico* study was conducted depending on the bacteriocins synthesized from the strains of *B. cereus* after a comprehensive literature review and database mining. According to the STRING dataset library (https://string-db.org/) of protein networking, a group of nine genes was found to be involved in biosynthesizing diversified bacteriocins (Fig. 4). The genes acting as the interaction source of the string networks are DJ87_3435, DJ87_3436, and DJ87_3437. However, DJ87_3435 mainly encodes an undifferentiated bacteriocin (Fig. 4). Among the bacteriocin genes, three uncharacterized peptides are encoded by the genes identified as DF87_1184 (*), DJ87_2735 (***), and DJ87_3435 (##) in Figure 4. The genes for radical SAM protein (**); cyclodehydratase domain (###); and SagB-type dehydrogenase domain protein (β*) of *B. cereus* are significantly regulated by each other (Fig. 5). In this research, two different gene clusters are observed. According to the “kmeans clustering,” DJ87_238 and DJ87_894 genes form cluster 1, while DJ87_1184, DJ87_3435, DJ87_3436, DJ87_3437, DJ87_3438, and sucB genes are involved in cluster 2 [32]. During the protein clustering process of *B. cereus*, the uncharacterized proteins (*, ***, ##) established vigorous clusters with a radical SAM protein (**); while the cyclodehydratase domain (###) strongly interacts with the SagB-type dehydrogenase domain protein (β*). Surprisingly, the proteins encoded by the DJ87_3436 and DJ873437 genes are comparatively distant-related to the rest of the bacteriocins (Fig. 5).

### Phylogenetic clustering of B. cereus bacteriocins

According to the BACTIBASE, *B. cereus* strains produce a few selective bacteriocins and antimicrobial peptides [29], among which “cerein 7” is the most importantly certified one for use for therapeutic purposes and new drug formulation [64]. In this study, it is phenomenal that cerein 7 possesses a broad spectrum of evolutionary relationships with the other bacteriocins synthesized from the *B. cereus* strains (Fig. 5A). It has been observed that cerein 7B has strong evolutionary footprints with the α/β hydrolase; cytotoxin K; hemolysin; oxidoreductase; tyrosine-type integrase; and NADH-quinone-oxidoreductase-C proteins of other *Bacillus* spp. (Fig. 5B). Unlike these proteins, cerein 7 is also connected in evolutionary footprints with some other antimicrobial peptides, which include NHE-A, NHE-H, and response-regulator-transcription factor proteins (Fig. 5B). Along with *B. cereus*, all of these bacteriocins from different *Bacillus* strains can be highly important in promoting immunological feedback against pathogenic diseases [65]. Antimicrobial peptides are reported to have significant effects in suppressing microbial infections and the ROS-mediated metabolism of cancer stem cells [66], where they may be synthesized as novel AMPs for experimental applications [67,68].

## Conclusion

In this research, *B. cereus* LOCK 1002 was isolated from yogurt samples, identified using 16S rRNA sequencing, and characterized physicochemically and biochemically in a fully controlled aseptic environment. The antimicrobial properties of the CFS of the newly identified LOCK 1002 strain against selective enteric pathogens were conducted following its consecutive antibiotic and antifungal sensitivity tests. The results showed that the LOCK 1002 strain is very susceptible to the antibiotics used. It was found to be resistant to the antifungal agents, Nystatin and Fluconazole. The antimicrobial activity of LOCK 1002 resulted in a very significant increase, making it a strong opportunistic antimicrobial candidate among all the *Bacillus* members. Finally, the *in silico* prediction and characterization of the gene clusters responsible for the antimicrobial activities were done considering their gene string network stability, interaction sources, CO, H, GF, DA, combined scores, and neighbor-joining profiles. A total of nine gene clusters were found to be associated with the synthesis of bacteriocins, and the chances of having cerein 7B in the CFS of LOCK 1002 were similar to the others, which could lead to exploring its clinical implication and establishing it as a therapeutic candidate in the coming days. Besides, it has been strongly predicted that there would be some undefined proteins clustering with the other functional bacteriocins of *B. cereus*.

## List of abbreviations

CFS, cell-free supernatant; LAB, lactic acid bacteria; MIC, minimum inhibitory concentration, PEMBA, Polymyxin pyruvate egg-yolk mannitol–bromothymol blue agar; PCR, polymerase chain reaction; ROS, Reactive oxygen species.
